# Prevalence and Risk Factors of Anemia Among Ethiopian Cancer Patients Undergoing Treatment: A Systematic Review and Meta‐Analysis

**DOI:** 10.1155/anem/7191757

**Published:** 2026-06-23

**Authors:** Frezer Kedir, Mathewos Mekonnen Gemmech, Yohannes Godie Ashebir, Mesfin Dabasa Jigi, Teferi Babu Itana, Ashenafi Tesfaye Yadesa, Dawit Tesfaye Rundasa, Aliyi Benti Daba, Sadik Abdulwehab

**Affiliations:** ^1^ School of Nursing, Jimma University, Jimma, Southwest Oromia, Ethiopia, ju.edu.et; ^2^ Department of Pediatric Nursing, Menelik II Medical and Health Science College, School of Nursing and Midwifery, Addis Ababa, Ethiopia; ^3^ Department of Neonatal Nursing, Menelik II Medical and Health Science College, School of Nursing and Midwifery, Addis Ababa, Ethiopia; ^4^ Department of Public Health, Institute of Health Sciences, Wollega University, Nekemte, Ethiopia, wollegauniversity.edu.et; ^5^ Department of Biomedical Science, Institute of Health Sciences, Wollega University, Nekemte, Ethiopia, wollegauniversity.edu.et; ^6^ Department of Surgical and Operating Theatre Nursing, Institute of Health Sciences, Wollega University, Nekemte, Ethiopia, wollegauniversity.edu.et; ^7^ Department of Surgical Nursing, Menelik II Medical and Health Science College, School of Nursing and Midwifery, Addis Ababa, Ethiopia; ^8^ School of Nursing, Wollega University, Nekemte, Oromia, Ethiopia, wollegauniversity.edu.et

**Keywords:** anemia, associated factors, cancer, Ethiopia, treatment

## Abstract

**Introduction:**

Anemia is a prevalent but under‐recognized cancer complication, particularly in low‐resource countries like Ethiopia. It significantly impacts treatment outcomes, fatigue, quality of life, and mortality rates. This review aims to estimate the prevalence of anemia and its risk factors among cancer patients in Ethiopia for clinical decision‐making and public health strategies.

**Methods:**

This study employed a systematic review and meta‐analysis design, sourcing evidence from various electronic databases until August 03, 2025. The data were extracted from June 01 to 30 and analyzed from July 01 to 25, with report generation till August 03, 2025, using R software. Meta‐analysis was performed using a random‐effects model, with forest plots illustrating pooled prevalence and associated factors. Heterogeneity was assessed using the I^2^ statistic, and study quality was evaluated using a validated tool.

**Results:**

Eleven studies involving diverse cancer populations across Ethiopia were included. The pooled prevalence of anemia among cancer patients was 39.5% (95% CI: 26.9%–52.1%). Advanced cancer stage, female sex, older age, poor nutritional status, rural residence, presence of comorbidities, hematologic malignancies, ≥ six cycles of chemotherapy, bone metastasis, and history of bleeding were factors associated with the development of cancer in patients who received treatment.

**Conclusion:**

Anemia is common among Ethiopian cancer patients, driven by both disease‐ and treatment‐related factors. Routine screening and targeted interventions are needed to improve outcomes.

**Highlight**
• What is already known?
◦ Anemia is a common complication in cancer patients, particularly during and after treatment.
◦ It negatively affects treatment effectiveness, quality of life, and survival outcomes.
◦ Risk factors such as advanced cancer stage, chemotherapy, and comorbidities contribute to anemia in cancer care.
• What does this paper add to the existing knowledge?
◦ This is the first systematic review and meta‐analysis to estimate the national pooled prevalence of anemia among Ethiopian cancer patients.
◦ It identifies various modifiable associated factors, including nutritional status, rural residence, and bleeding history.
◦ The study highlights the urgent need for standardized anemia screening and targeted interventions in Ethiopia’s oncology settings.

## 1. Introduction

The Global Cancer Observatory reported that cancer remains a major global public health challenge, with an estimated 19.3 million new cases and 10 million deaths in 2020, a burden that is projected to continue rising, particularly in low‐ and middle‐income countries (LMICs) [1]. Ethiopia, like many LMICs, is experiencing an increasing cancer burden driven by population aging, lifestyle transitions, and limited access to early detection and comprehensive treatment services [2].

Anemia is one of the most frequent hematological complications among cancer patients, particularly those receiving chemotherapy. It results from multifactorial mechanisms, including bone marrow suppression, cancer‐related blood loss, inflammation, and impaired nutrient utilization [3, 4]. The prevalence of anemia among cancer patients receiving treatment varied across different countries, as 41.1% in Thailand [5], 50.5% in Saudi Arabia [6], 48.1% in Spain [7], and 28% in Italy [8]. In Ethiopia, reported prevalence ranges widely from 23% to 75.4%, indicating substantial heterogeneity across studies [9–19].

Anemia is a common yet often overlooked complication among cancer patients undergoing treatment, presenting significant health challenges [20]. Despite its high prevalence, anemia is often poorly managed in cancer care due to factors such as gender, age, stage, and treatment type, highlighting the need for improved screening, early diagnosis, and effective management strategies [15, 19].

Cancer‐related anemia is clinically important as it is associated with fatigue, reduced cognitive function, impaired quality of life, and decreased treatment tolerance. Studies have also reported increased mortality risk among anemic cancer patients, highlighting its prognostic significance [21, 22]. Despite this, anemia remains under‐recognized and inconsistently managed in oncology care, partly due to variability in screening practices, delayed diagnosis, and limited provider awareness, particularly in resource‐constrained settings [23]. Additionally, anemia is often under‐managed in low‐resource areas due to inadequate provider education, standardized treatment protocols, and limited access to essential therapies, necessitating urgent improvements in screening, early diagnosis, and accessible treatment options. In low‐resource countries, management of cancer‐related anemia is further challenged by limited access to recommended therapies such as erythropoiesis‐stimulating agents and intravenous iron, as well as inadequate implementation of standardized treatment protocols [24]. In addition, the psychosocial burden of anemia, especially fatigue and reduced functional status, is often insufficiently addressed in routine cancer care [4, 25].

Despite the known burden and consequences of cancer‐related anemia, there is currently no synthesized national‐level evidence regarding its prevalence and associated risk factors in Ethiopia. This lack of consolidated data limits the ability of policymakers and healthcare providers to design targeted interventions and optimize supportive care for cancer patients. Conducting a systematic review is essential to pool existing findings, quantify the magnitude of anemia, and identify contributing factors among cancer patients undergoing treatment in Ethiopia. This review addresses a critical knowledge gap in the management of anemia among cancer patients and supports the development of national guidelines and clinical decision‐making tools tailored to cancer care pathways. This systematic review and meta‐analysis aim to estimate the pooled prevalence of anemia among cancer patients in Ethiopia and identify factors associated with its occurrence. The review intends to provide evidence to support clinical decision‐making, optimize supportive care for oncology patients, and guide future research in the context of cancer‐related anemia in Ethiopia.

## 2. Methods

### 2.1. Study Design

The review follows a systematic review and meta‐analysis design, conducted in line with the Preferred Reporting Items for Systematic Reviews and Meta‐Analyses (PRISMA 2020) guidelines [26]. A structured and replicable methodology was applied to identify, appraise, and synthesize the existing studies reporting on anemia prevalence and its predictors among cancer patients in Ethiopia. This systematic review and meta‐analysis were conducted and reported in accordance with the PRISMA guidelines, and no amendments were made to the protocol.

### 2.2. Research Questions

The central question addressed by this review is: What is the pooled prevalence of anemia among cancer patients in Ethiopia? What are the major related factors associated with anemia in these patients that could inform public health interventions and early cancer detection programs?

### 2.3. Review Protocol

To ensure accountability and reduce potential bias, a detailed review protocol was developed before commencing the review. The protocol outlined the review objectives, eligibility criteria, search strategy, data extraction procedures, critical appraisal framework, and synthesis method. It was designed by the Joanna Briggs Institute (JBI) Manual for Evidence Synthesis [27], which provides methodological guidance for qualitative reviews. The protocol was registered with PROSPERO under Registration No.: CRD420251045327 on April 20, 2025, serving as a formal commitment to methodological integrity.

### 2.4. Search Strategy

A comprehensive and systematic search was conducted in the following electronic databases: PubMed, Scopus, Web of Science, CINAHL, African Journals Online (AJOL), and Google Scholar, along with Ethiopian university repositories for the gray literature. The selected databases were chosen due to their relevance to biomedical, clinical, and nursing research, and their comprehensive coverage of peer‐reviewed literature relevant to the study topic. The search strategy combined Medical Subject Headings (MeSH) and free‐text terms such as “Anemia” OR “anemia,” “Cancer” OR “malignancy” OR “oncology,” “Prevalence” OR “burden,” “Associated factors” OR “risk factors,” “Ethiopia.” Boolean operators (AND/OR) were used to refine the results. The detailed database‐specific search strings used in this review are provided in Supporting Appendix 1.

**FIGURE 1:**
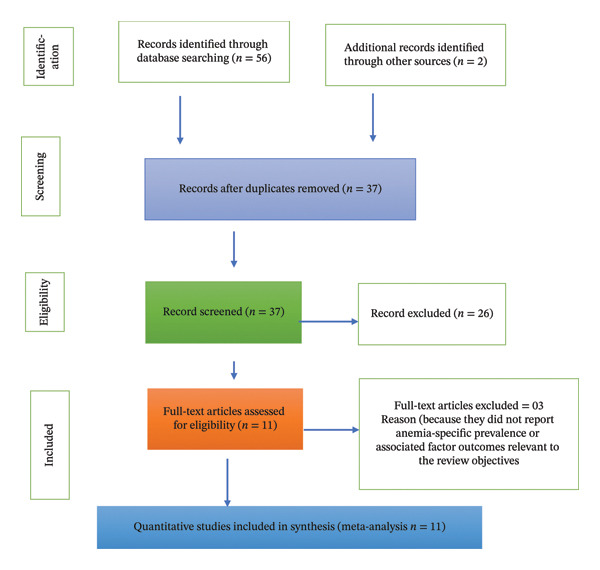
PRISMA 2020 flow diagram showing the selection process of studies included in the systematic review and meta‐analysis on anemia among cancer patients in Ethiopia, 2025.

Reference lists of relevant articles were also manually screened for additional studies. Searches included all publications up to May 03, 2025. The data were extracted from June 01 to 30 and analyzed from July 01 to 25, with report generation till August 03, 2025 (Figure 1).

### 2.5. Inclusion and Exclusion Criteria

Studies were included if they met the following criteria: (1) conducted among cancer patients in Ethiopia; (2) reported the prevalence of anemia and/or analyzed factors associated with anemia; (3) used observational study designs, including cross‐sectional, cohort, or case–control studies; (4) were peer‐reviewed articles, academic theses, dissertations, or relevant gray literature; (5) were published in English; and (6) provided sufficient data to extract or calculate anemia prevalence and/or associated effect estimates.

Studies were excluded if they were case reports, narrative reviews, systematic reviews, editorials, commentaries, or conference abstracts. In addition, studies with incomplete data, or those that did not report anemia‐specific outcomes or sufficient information for data extraction, were excluded from the analysis.

### 2.6. Search Outcomes

A total of 56 records were identified through database searching: PubMed [13], Scopus [11], Web of Science [10], Google Scholar [16], and AJOL [6], and two additional records were identified through other sources, resulting in 58 records before screening. All identified studies were imported into EndNote for reference management and duplicate removal. Two reviewers independently screened titles and abstracts against the inclusion criteria. Full texts of eligible studies were retrieved and assessed. Discrepancies between reviewers were resolved through consensus or consultation with a third reviewer. After removing duplicates and screening abstracts and full texts, eleven studies met the inclusion criteria and were included in the final review. The PRISMA flow diagram summarizes the study selection process, from identification to inclusion (Figure 1).

### 2.7. Data Extraction

Data were extracted using a standardized data abstraction form, developed based on the JBI methodology. Extracted variables included the following: study characteristics: author(s), publication year, study region, design, and setting; population details: sample size, age, sex, type of cancer, and stage of disease; anemia‐specific data: prevalence of anemia, criteria used for diagnosis, severity classification, and associated factors (e.g., nutritional status, chemotherapy, comorbidities); and statistical outcomes: crude and adjusted odds ratios (ORs), confidence intervals (CIs), and *p* values for associated factors. Anemia definitions varied across studies and were taken as reported by the original authors (Table 1).

**TABLE 1:** Characteristics of included studies on the prevalence of anemia and its associated factors among cancer patients in Ethiopia, a systematic review and meta‐analysis.

Author, year	Title	Region/setting	Design	Sample and population	Population details	Cancer services used	Prevalence of anemia (95% CI)	Associated factors (AOR, CI, *p* value)	Limitations	Conclusions	Recommendations
Aynalem et al., 2022	Hematological abnormalities before and after initiation of cancer treatment among breast cancer patients	Not specified (likely Gondar)	Retrospective comparative study	Breast cancer patients (exact *n* not specified)	Women with confirmed diagnosis; pretreament/post‐treatment data collected	Chemotherapy ± surgery	26.4% (95% CI: 21.3, 31.5) after the initiation of cancer treatment	Decline in hemoglobin and RBC levels post‐treatment. Factors not statistically modeled	Small sample size; lacks multivariate analysis	Chemotherapy reduces hemoglobin and increases the risk of anemia	Need for preventive and supportive care during cancer treatment
Belachew et al., 2016	Pattern of chemotherapy‐related adverse effects among adult cancer patients	Gondar University Referral Hospital	Cross‐sectional	203 cancer patients with ADRs	Mean age: 43.3 years; 58.6% female; hematologic and breast cancer common	Chemotherapy (mono‐ and polychemotherapy)	Overall: 9.3% anemia among ADRs (76 cases of anemia)	Not explicitly analyzed in regression, but age, number of drugs, and dose were linked to ADR severity	No control group; focus was on ADRs, not anemia specifically	Anemia is a common ADR of chemotherapy, often severe	Strengthen pharmacovigilance and ADR monitoring in oncology settings.
Berta et al., 2024	Hematological changes in women with cervical cancer before and after cancer treatment	University of Gondar	Retrospective cohort	219 cervical cancer patients	Median age: 50 years; 71.3% early stage; 11.4% HIV+	Chemo‐radiotherapy, cryotherapy, and surgery	Before: 24.6% (95% CI: 18.7–30.6) After: 44.3% (95% CI: 37.9–50.7)	Late‐stage (AOR = 7.6, CI: 3.7–15.5) HIV+ (AOR = 2.3, CI: 1.1–6.2) rural residence (AOR = 2.5, CI: 1.14–5.5)	Single‐center, retrospective, limited to women	Anemia increased significantly post‐treatment. Risk factors included advanced stage, HIV status, and rural residence.	Routine hematologic monitoring pre‐ and post‐treatment; prioritizing high‐risk patients.
Kassie et al. (2025)	Differences in the count of blood cells pre‐ and postchemotherapy in patients with cancer	Mekele Comprehensive Specialized Hospital, Northern Ethiopia	Retrospective	354 patients who started chemotherapy from 2018 to 2022	Adults with stage III or IV cancer, both sexes	Chemotherapy (Phase IV)	Pre‐chemotherapy: 25.14% (95% CI: 20.88–29.94); postchemotherapy: 35.54% (95% CI: 30.75–40.73)	Not explicitly stated with AORs; findings presented by *t* test and % changes.	No control group; limited generalizability beyond one hospital	Chemotherapy significantly reduces hematological parameters, including Hb, RBC, WBC, and platelets	Replacement therapy should be considered postchemotherapy
Kifle et al. (2019)	Prevalence of anemia and associated factors among newly diagnosed patients with solid malignancy	Tikur Anbessa Specialized Hospital, Addis Ababa, Ethiopia	Cross‐sectional	422 newly diagnosed patients with solid malignancies	Median age: 45; 66% female; various tumor types, mostly gynecologic and breast	Diagnostic (no treatment started)	23%	ECOG performance status (AOR = 3.344, 95% CI: 1.410–7.927); bleeding history (AOR = 3.628, 95% CI: 1.800–7.314), *p* < 0.05	Lack of treatment for most anemic patients; only the pretreatment stage was assessed	Anemia is common at baseline; high prevalence in gynecologic and colorectal cancers	Need for early anemia screening and treatment before cancer therapy
Setegn and Nakie (2024)	Baseline anemia and its associated factors among adult cancer patients	Northwest Amhara Regional State Referral Hospitals, Ethiopia	Cross‐sectional	315 adult cancer patients from two oncology units	Adults ≥ 18, various cancer types, both sexes, treatment‐naïve	Diagnostic (baseline assessment)	34.84%	Female sex (AOR = 1.97; 95% CI: 1.00–3.87), Underweight (AOR = 1.96; 95% CI: 1.09–3.52), Stage III cancer (AOR = 2.35; 95% CI: 1.12–3.01)	Cross‐sectional limits causality; BMI and stage self‐reported from the chart	High burden of baseline anemia, especially in women, underweight, and advanced‐stage patients	Target interventions for women, underweight, and Stage III patients to manage anemia early
Wassie et al. (2021)	Prevalence and associated factors of baseline anemia among cervical cancer patients in Tikur Anbesa Specialized Hospital, Ethiopia	Tikur Anbessa Specialized Hospital, Ethiopia	Retrospective chart review (census)	634 medical records of cervical cancer patients from Jan 2014–Dec 2016	Women with cervical cancer, mean age 49.82, Stage I–IV, various sociodemographic profiles	Diagnostic stage (pretreatment)	50.95% (95% CI: 47.3–54.7)	Stage IV cancer (AOR = 2.38, 95% CI: 1.21–4.67, *p* = 0.012); substance use (AOR = 2.03, 95% CI: 1.21–3.41, *p* = 0.007); comorbidity (AOR = 3.32, 95% CI: 2.25–4.90, *p* < 0.001); being divorced (protective, AOR = 0.6, 95% CI: 0.36–0.98, *p* = 0.042)	Retrospective chart data may be incomplete; data from 2014 to 2016 may not reflect current trends; single‐center only.	High prevalence of anemia; advanced stage, substance use, and comorbidities increase risk; being divorced is protective.	Prioritize screening/treatment of anemia in advanced‐stage patients, substance users, and those with comorbidities; expand cervical cancer early detection efforts.
Wassie et al., 2024	Baseline anemia and its associated factors among adult cancer patients at Northwest Amhara Regional State Referral Hospitals, Ethiopia	Northwest Amhara, Ethiopia (UoGCSH, FHCSH)	Institutional‐based cross‐sectional	310 adult cancer patients	Mean age: 45.8; 70.3% female; various cancer types	Oncology treatment units	34.84% (95% CI: 29.71–40.34)	Female (AOR = 1.97, CI: 1.00–3.87, *p* < 0.05); underweight (AOR = 1.96, CI: 1.09–3.52); stage III cancer (AOR = 2.35, CI: 1.12–3.01)	Cross‐sectional design limits causal inference; location‐specific	High prevalence; more common in females, underweight, and advanced‐stage patients	Special attention to high‐risk groups for anemia management
Woldemariam et al., 2023	Prevalence and associated factors of anemia among people with cancer in ACSH, Tigray, Ethiopia	Ayder Comprehensive Specialized Hospital (ACSH), Tigray, Ethiopia	Institutional‐based cross‐sectional	72 adult cancer patients	Mean age: 47; 54.2% male	Oncology clinic; chemotherapy treatment	45.8%	Unemployed (OR = 2.17, CI: 1.09–4.29, *p* = 0.027); rural (OR = 3.75, CI: 1.25–11.30, *p* = 0.019); stage III/IV (OR = 10.77, CI: 3.58–32.41, *p* < 0.001); Duration ≥ 6 months (OR = 3.54, CI: 1.29–9.73, *p* = 0.014); High chemocycles (OR = 3.00, CI: 1.09–8.25, *p* = 0.033)	Small sample size; single institution; excluded critical patients	High anemia prevalence; multiple socioeconomic and clinical factors involved	Screen and manage anemia in rural, unemployed, late‐stage patients
Wondimneh et al., 2021	Comparison of hematological and biochemical profile changes in pre‐ and postchemotherapy treatment of cancer patients	Ayder Comprehensive Specialized Hospital, Mekelle, Ethiopia	Retrospective cohort	376 cancer patients	60.6% female; common cancers: breast, lymphoma, sarcoma	Chemotherapy (varied regimens)	Not reported as prevalence; hemoglobin significantly decreased postchemotherapy	Chemotherapy is associated with significant reductions in Hb, RBC, WBC, and PLT (*p* < 0.001)	No specific AOR; lacked individual anemia determinants	Chemotherapy significantly reduces hematological parameters	Monitor and manage hematologic profiles before and after chemotherapy
Wondm et al. (2024)	Incidence and associated factors of chemotherapy‐induced anemia among adult cancer patients in Northwest Ethiopia	Felegehiwot & University of Gondar Comprehensive Specialized Hospitals, Northwest Ethiopia	Retrospective follow‐up study	402 adult cancer patients with baseline Hb ≥ 12 g/dL undergoing first‐time chemotherapy (2019–2021)	Age ≥ 18, no baseline anemia, variety of solid and hematologic cancers	Chemotherapy (21 regimens over multiple cycles)	75.4% (95% CI: 70.7–79.8) experienced CIA	Age ≥ 60 (AOR = 1.8; 95% CI: 1.4–3.5; *p* = 0.043); hematologic cancer (AOR = 3.7; 95% CI: 3.2–5.7; *p* = 0.021); obesity (AOR = 3.4; 95% CI: 2.3–6.9; *p* = 0.028); ≥ 6 cycles (AOR = 3.8; 95% CI: 3.2–5.1; *p* = 0.019); bone metastasis (AOR = 2.9; 95% CI: 1.2–4.7; *p* = 0.025)	No national registry; no molecular data; chemotherapy dose intensity not captured	CIA is highly prevalent, particularly in older, obese patients, those with hematologic malignancies, bone metastases, or extended treatment	Early screening and targeted monitoring of high‐risk patients is essential; consider anemia management protocols during chemotherapy.

### 2.8. Quality Appraisal

The quality and risk of bias of the included studies were assessed using the JBI critical appraisal checklists appropriate to each study design. Two reviewers (SA and FK) independently performed the quality assessments. Inter‐rater agreement between the two reviewers during study selection and quality appraisal was assessed using Cohen’s kappa statistic, which demonstrated substantial agreement. The Cohen’s kappa values were 0.82 for study selection and 0.79 for quality appraisal, indicating substantial agreement. Studies scoring ≥ 7 were categorized as high quality, 4–6 as moderate, and ≤ 3 As low quality. Only studies of moderate to high quality were included in the meta‐analysis (Table 2).

**TABLE 2:** JBI critical appraisal on anemia among cancer patients in Ethiopia, 2025.

Study no.	Aynalem et al., 2022	Belachew et al., 2016	Berta et al., 2024	Kassie et al. (2025)	Kifle et al. (2019)	Setegn & nakie (2024)	Wassie et al. (2021)	Wassie et al., 2024	Woldemariam et al., 2023	Wondimneh et al., 2021	Wondm et al. (2024)
Was there a clear statement of the aims of the research?	✔	✔	✔	✔	✔	✔	✔	✔	✔	✔	✔
Was the study design appropriate for the aims of the research?	✔	✔	✔	✔	✔	✔	✔	✔	✔	✔	✔
Was the sample representative of the population studied?	✔	✘	✔	✔	✔	✘	✔	✔	✔	✔	✘
Was the sample size adequate?	✔	✘	✔	✔	✔	✔	✔	✔	✔	✔	✔
Were the study subjects and the setting described in detail?	✘	✔	✔	✔	✔	✔	✔	✔	✔	✔	✔
Was the data collected reliably?	✔	✔	✔	✘	✔	✔	✔	✔	✘	✔	✔
Were the statistical analyses used to assess the data appropriate?	✔	✔	✔	✔	✔	✘	✘	✔	✔	✔	✘
*Were the findings valid and applicable to the local context?*	✔	✔	✔	✔	✔	✔	✔	✔	✔	✔	✔
*Overall Quality*	7	6	8	7	8	6	7	8	7	8	6

*Note:* ✔ = yes (criterion met), ✘ = no (criterion not met), and ? = unclear (not adequately reported).

### 2.9. Data Synthesis and Statistical Analysis

Meta‐analyses were conducted using R software (Version 4.0). A random‐effects model was employed to calculate the pooled prevalence of anemia and summary effect sizes for associated factors, due to expected heterogeneity among studies. Prevalence estimates were reported with 95% CIs. Heterogeneity was assessed using I^2^ statistics, with values > 50% indicating substantial heterogeneity. Subgroup analyses were conducted based on region, cancer type, study setting, and anemia definition. Meta‐regression was used to explore potential sources of heterogeneity. Publication bias was evaluated through funnel plots and Egger’s regression test. Sensitivity analyses were performed by excluding studies with small sample sizes or high risk of bias to assess the robustness of the findings.

### 2.10. Ethical Considerations

This study involved a review of existing published and publicly accessible data, and thus, ethical approval was not required. However, all included studies were reviewed to ensure that they had obtained ethical clearance. Proper citation of all sources was maintained, and data were used solely for academic purposes.

## 3. Result

The included studies were conducted across diverse regions of Ethiopia and featured both institutional‐ and community‐based designs [9–19]. All eleven studies employed observational designs, primarily cross‐sectional [10, 13, 14, 16–18]. Retrospective cohort or follow‐up designs [11, 12, 19] were applied to assess hematological changes before and after treatment. Aynalem et al. [9] used a retrospective comparative approach to examine anemia before and after breast cancer therapy. All studies relied on secondary data collected from medical records or institutional databases.

The included studies were conducted in various regions of Ethiopia, covering both urban and regional oncology centers. Major hospital‐based studies were carried out at Tikur Anbessa Specialized Hospital in Addis Ababa [13, 15], University of Gondar Referral Hospital [10, 11, 19], Felege Hiwot Referral Hospital in Bahir Dar [16], and Ayder Comprehensive Specialized Hospital in Mekelle [17, 18]. Most studies were institutional, offering a fairly broad view of cancer‐related anemia across Ethiopia’s main oncology care centers.

Sample sizes varied widely, ranging from as low as 72(17) to as high as 634 participants [19]. All studies focused on evaluating the prevalence of anemia and its association with demographic, clinical, and treatment‐related factors [9–19].

The studies include diverse populations of cancer patients, with a range of cancer types and stages [9–19]. Most studies involve both sexes, and patient ages span from early adulthood to older age, with several studies targeting patients in advanced cancer stages or those undergoing chemotherapy or other treatments.

The studies reviewed indicate that a range of cancer services are utilized by patients in Ethiopia, with chemotherapy being the most frequently employed treatment modality across various cancer types [9, 12, 16, 18, 19]. Some studies specifically highlighted the use of combined treatments such as chemotherapy and radiotherapy [11]. Surgical interventions combined with chemotherapy [9, 11]. In addition, less common procedures like cryotherapy were identified as treatment options for early stage cervical cancer [11].

### 3.1. Prevalence of Anemia Among Cancer Patients Receiving Treatment

**FIGURE 2:**
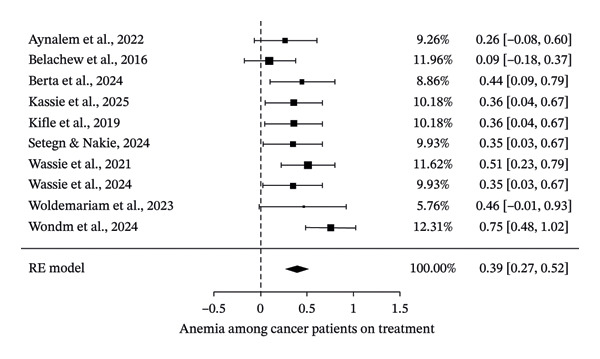
Forest plot illustrating the pooled prevalence of anemia among cancer patients in Ethiopia included in the systematic review and meta‐analysis, 2025.

The prevalence of anemia among cancer patients varies widely across studies, ranging from 23% to 75.4%. Baseline anemia rates are also notable, with some studies reporting around 24.6%–50.95% prevalence before treatment [11, 15]. The pooled prevalence of anemia was calculated based on the definitions reported in the original studies. Each included study applied its own hemoglobin cutoff values to define anemia, with some using WHO criteria (< 12 g/dL for women and < 13 g/dL for men), while others used study‐specific thresholds for baseline anemia, chemotherapy‐induced anemia, or post‐treatment anemia. These differences were not harmonized across studies, and no subgroup analysis was performed due to the limited number of studies within each anemia definition category. Therefore, the pooled prevalence should be interpreted with caution as an overall summary estimate reflecting heterogeneous definitions of anemia across studies. The overall pooled prevalence was found to be 39.5% (95% CI: 26.9%–52.1%) (Figure 2).

The heterogeneity analysis in this meta‐analysis revealed moderate variability among the included studies. The between‐study variance (Tau^2^) was estimated at 0.0144 with a standard error of 0.0192, and the corresponding Tau value was 0.120. The *I* ^2^ statistic, which indicates the percentage of total variation across studies due to heterogeneity rather than chance, was 35.4%. The moderate heterogeneity observed across studies may be explained by methodological and clinical differences among the included studies, including variations in anemia definitions and diagnostic thresholds, differences in study design, diversity in cancer types and stages, treatment modalities, and regional healthcare settings. These differences likely contributed to the variability in prevalence estimates and justified the application of a random‐effects model. Overall, the moderate level of heterogeneity supports the use of a random‐effects model, which accounts for between‐study variability while providing a more generalized estimate of the pooled prevalence.

**FIGURE 3:**
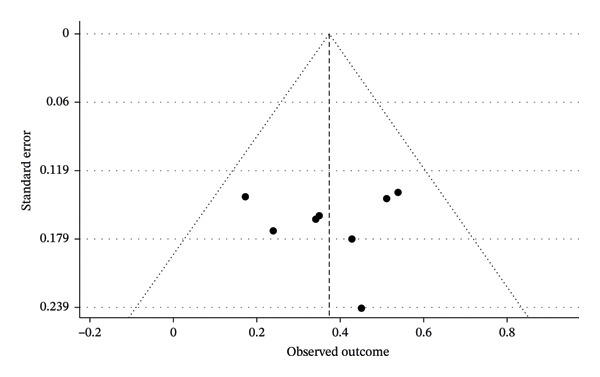
Funnel plot assessing publication bias for studies included in the meta‐analysis of the pooled prevalence of anemia among cancer patients in Ethiopia, 2025.

The funnel plot analysis, used to assess publication bias, was supported by multiple statistical tests. Kendall’s Tau test (*τ* = −0.163, *p* = 0.525) and Egger’s regression test (intercept = −0.221, *p* = 0.825) both indicated no statistically significant asymmetry in the funnel plot. These findings suggest that there is no strong evidence of publication bias in the included studies on the prevalence of anemia among cancer patients after the initiation of treatment. Therefore, the funnel plot likely appears symmetric, supporting the reliability of the pooled prevalence estimate (Figure 3).

### 3.2. Sensitivity Analysis

A sensitivity analysis was conducted to evaluate the robustness of the pooled prevalence estimate of anemia among cancer patients after the initiation of treatment. This process involved systematically removing one study at a time and observing the impact on the overall effect size and heterogeneity. When each study was excluded one at a time, the pooled prevalence estimate fluctuated slightly, but no single study markedly changed the direction or magnitude of the overall effect size. The I^2^ value remained within a moderate range (approximately 30%–40%), indicating that the heterogeneity did not significantly decrease or increase after removing individual studies. These findings suggest that the pooled estimate is not overly influenced by any single study, confirming the stability and reliability of the meta‐analysis results (Table 3).

**TABLE 3:** Influence of each study on pooled effect size on anemia among cancer patients in Ethiopia, 2025.

Study excluded	Pooled prevalence	95% CI	*I* ^2^ (%)	Change observed
Aynalem et al., 2022	0.401	0.273–0.528	34.2	No significant change
Belachew et al., 2016	0.409	0.280–0.538	35.8	No significant change
Berta et al., 2024	0.382	0.251–0.513	36.1	No significant change
Kassie et al., 2025	0.387	0.258–0.517	34.9	No significant change
Kifle et al., 2019	0.389	0.260–0.518	36.5	No significant change
Setegn & Nakie, 2024	0.397	0.267–0.526	35.0	No significant change
Wassie et al., 2021	0.379	0.249–0.508	36.2	No significant change
Wassie et al., 2024	0.396	0.266–0.525	34.8	No significant change
Woldemariam et al., 2023	0.393	0.265–0.521	34.0	No significant change
Wondm et al., 2024	0.371	0.241–0.500	36.3	No significant change

Factors associated with the development of anemia among cancer patients receiving treatment.

**FIGURE 4:**
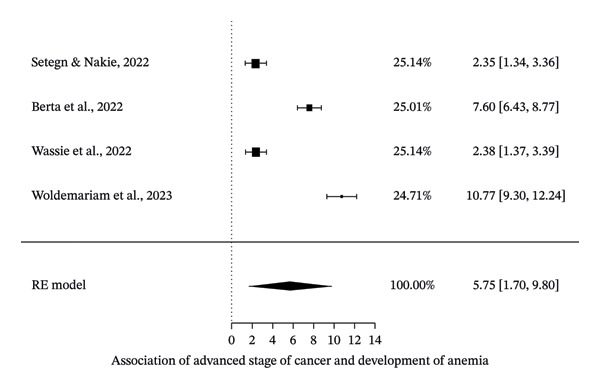
Forest plot shows the association between advanced stages of cancer and development of anemia among cancer patients who received treatment in Ethiopia, a systematic review and meta‐analysis, 2025.

The reviewed article showed that the advanced stage of cancer showed a significant association with the development of anemia among cancer patients in Ethiopia. Advanced‐stage cancer was consistently identified as a risk factor across multiple studies [11, 14, 15, 17]. The pooled effect size for the association between advanced‐stage cancer and the development of anemia was found to be 5.75 (95% CI: 1.7–9.8), indicating that individuals living with advanced‐stage disease were more than six times as likely to experience the development of anemia among cancer patients in Ethiopia than early stage disease (Figure 4).

**FIGURE 5:**
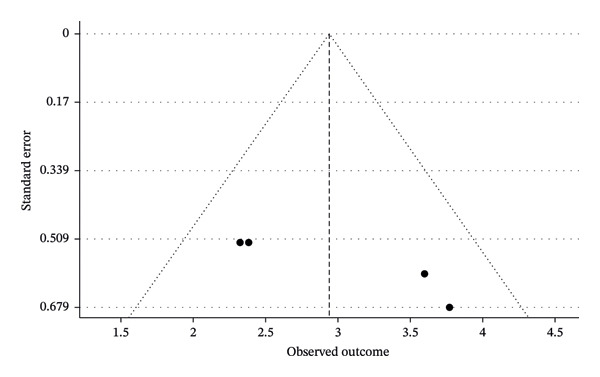
Funnel plot shows the association between advanced stages of cancer and development of anemia among cancer patients who received treatment in Ethiopia, a systematic review and meta‐analysis, 2025.

The meta‐analysis revealed moderate heterogeneity among the included studies, with an I^2^ of 42.85% and Tau^2^ of 0.2441 (SE = 0.4664). Regarding publication bias, the funnel plot appeared relatively symmetrical, suggesting minimal potential bias (Figure 5).

One study found that gender was significantly associated with the development of anemia among cancer patients who received treatment. Females were two times more likely to develop anemia after the initiation of the treatment than male patients (OR = 1.97; 95% CI: 1.00–3.87) [14].

Another study showed that older age was also associated with a higher risk of anemia. Patients aged 60 years and above had two times greater odds of developing anemia compared to those aged under 60 years (AOR = 1.8; 95% CI: 1.4–3.5) [19].

Two studies showed that there is an association between nutritional status and the development of anemia among cancer patients who received treatment [14, 19]. Undernourished patients demonstrated significantly higher odds of anemia, as underweight individuals had nearly double the risk (AOR = 1.96; 95% CI: 1.09–3.52) [14], and obese patients had more than three times the odds (AOR = 3.4; 95% CI: 2.3–6.9) of developing anemia compared to those with normal nutritional status [19].

Two studies showed that there is a link between residence area and the development of anemia among cancer patients who received treatments [11, 17]. Berta et al. [11] reported that cancer patients living in rural areas had 2.5 times higher odds of developing anemia compared to urban residents (AOR = 2.50; 95% CI: 1.14–5.50; SE = 0.417). Similarly, Woldemariam et al. [17] found that those living in rural areas were 3.7 times more likely to develop anemia following cancer treatment than those living in urban areas (AOR = 3.75, 95% CI: 1.25–11.30).

One study showed that comorbid conditions were significantly associated with an increased risk of anemia among cancer patients [15]. Patients with comorbidities were over three times more likely to develop anemia than their counterparts (AOR = 3.32; 95% CI: 2.25–4.90; SE = 0.201).

Hematologic cancer was strongly associated with the development of anemia among cancer patients. As reported by Wondm et al. [19], individuals diagnosed with hematologic malignancies were 3.7 times more likely to develop anemia compared to those with other cancer types (AOR = 3.7; 95% CI: 3.2–5.7).

Chemotherapy‐related factors, specifically receiving six or more cycles of chemotherapy, were significantly associated with the development of anemia among cancer patients [17, 19]. In two independent studies, Woldemariam et al. [17] reported that those who took chemotherapy six or more times were three times more likely to develop anemia (AOR = 3.00, 95% CI: 1.09–8.25), and Wondm et al. [19] showed 3.8 times more likely to develop anemia (AOR = 3.80, 95% CI: 3.20–5.10).

The presence of bone metastasis was significantly associated with increased odds of developing anemia among cancer patients [19] as patients with bone metastases had nearly three times greater odds of developing anemia compared to those without metastasis (AOR = 2.9, 95% CI: 1.2–4.7). Finally, one study showed that having a history of bleeding was significantly associated with increased odds of developing anemia among cancer patients [13]. Patients with a history of bleeding are 3.6 times more likely to develop anemia compared to those who have no history of bleeding (AOR = 3.63; 95% CI: 1.80–7.31; SE = 0.366).

## 4. Discussion

This systematic review included eleven studies conducted across diverse geographic regions of Ethiopia, encompassing both institutional‐ and community‐based settings [9–19]. The pooled prevalence of anemia among cancer patients who received treatment was 39.5% (95% CI: 26.9–52.1), which is similar to the study done in Thailand, which accounts for 41.1% [5], 50.5% in Saudi Arabia [6], 48.1% in Spain [7], and 28% in Italy [8]. This is due to Anemia, a prevalent cancer issue, being influenced by chemotherapy’s myelosuppressive effects, advanced disease stages, nutritional deficiencies, chronic inflammation, chemotherapy regimens, dose reductions, and specific types, requiring routine screening for improved patient outcomes [28]. These international findings corroborate the significant prevalence of anemia among cancer patients undergoing treatment, emphasizing the need for routine screening and management strategies to improve patient outcomes and quality of life. The interpretation of the pooled prevalence of anemia should be made with caution due to variability in anemia definitions across the included studies. Although most studies applied WHO hemoglobin thresholds, others used different cutoff values or classified anemia based on clinical context, such as chemotherapy‐induced or post‐treatment anemia. This methodological heterogeneity may have influenced the pooled estimate and limited direct comparability across studies. In the absence of sufficient studies for subgroup analysis, we present an overall pooled estimate that should be considered an aggregated summary rather than a precise measure for a specific anemia category.

The reviewed article showed that the advanced stage of cancer showed a significant association with the development of anemia among cancer patients in Ethiopia [11, 14, 15, 17], which indicated that individuals living with the advanced stage of the disease were more than six times as likely to experience the development of anemia. The finding is similar to a study done in the United States [29], China [30], India [31], and Saudi Arabia [6]. The strong association between advanced‐stage cancer and anemia may be explained by multiple biological mechanisms, including bone marrow infiltration by malignant cells, chronic inflammation causing impaired iron metabolism through elevated inflammatory cytokines, nutritional deficiencies related to cancer cachexia, tumor‐associated bleeding, and cumulative myelosuppressive effects of chemotherapy and radiotherapy. These mechanisms collectively impair erythropoiesis and contribute to anemia progression among patients with advanced disease. However, the relatively wide CI (95% CI: 1.7–9.8) suggests some variability across studies, and therefore, the pooled estimate should be interpreted cautiously. Integrating early anemia screening and management into cancer care, particularly for advanced disease patients, can reduce the burden of anemia, improve overall outcomes, and enhance the quality of life for cancer patients.

The review showed that gender was significantly associated with the development of anemia among cancer patients who received treatment. Females were two times more likely to develop anemia after the initiation of the treatment than male patients [14]. This finding aligns with research from other countries in the United States [32], Saudi Arabia [33], Spain [7], and Europe [34]. The higher prevalence of anemia in females may be attributed to factors such as menstrual blood loss, lower baseline hemoglobin levels, nutritional deficiencies, and hormonal differences, which exacerbate the impact of cancer treatments like chemotherapy and radiotherapy. Gender‐sensitive anemia management strategies in cancer care, including regular screening and targeted interventions like iron supplementation and nutritional support, should be prioritized for female cancer patients to enhance treatment outcomes and prevent complications.

Another finding showed that older age is linked to a higher risk of anemia, with patients aged 60 years and above having twice the odds of developing anemia than their counterparts [19]. The finding is similar to a study done in the United States [29], Denmark [35], South Korea [36], Italy [37], Saudi Arabia [33], and Spain [7]. The risk of anemia in older adults is heightened by factors such as natural aging, cancer treatments, comorbid conditions, and nutritional deficiencies, which can exacerbate anemia due to a decline in bone marrow function and hematopoiesis. Healthcare providers should prioritize early anemia screening and management in older cancer patients, particularly those undergoing chemotherapy, to improve patient outcomes and quality of life.

The reviewed articles show that there is a linkage between nutritional status and the development of anemia in cancer patients receiving treatment, with undernourished patients having higher odds of anemia, overweight individuals having double the risk, and obese patients over three times more likely to develop anemia [14, 19]. This finding is similar to a study done in Spain [7], the United States [29], South Korea [36], Italy [37], Saudi Arabia [33], and Denmark [35]. The coexistence of increased anemia risk among both underweight and obese cancer patients highlights the complex relationship between nutritional status and hematologic health. Undernutrition may contribute to anemia through micronutrient deficiencies, poor dietary intake, cancer cachexia, and impaired erythropoiesis. Conversely, obesity may contribute through chronic low‐grade inflammation, elevated hepcidin production, impaired iron utilization, and obesity‐associated metabolic dysregulation. These findings suggest that both extremes of nutritional status may negatively affect hemoglobin production and iron metabolism in cancer patients. Therefore, regular nutritional screening and timely interventions such as individualized dietary support and supplementation are recommended to reduce anemia‐related complications and improve overall treatment outcomes in cancer care.

The review revealed that there is a linkage between residence area and the development of anemia among cancer patients who received treatments [11, 17] and reported that cancer patients living in rural areas were more likely to have odds of developing anemia compared to urban residents. The finding is in line with a study done in China [38], Brazil [39], Nigeria [40], and Saud Arabia [33]. Rural areas face increased cancer risk due to inadequate healthcare, delayed diagnosis, poor nutrition, and reduced health literacy, with obstacles such as transportation issues, inadequate infrastructure, and specialized care shortages potentially worsening anemia. Healthcare systems should improve early cancer screening, nutritional support, and regular anemia monitoring while prioritizing community outreach and health education.

The reviewed article reveals that cancer patients with comorbid conditions are at a significantly higher risk of developing anemia, with these patients being over three times more likely to develop the condition [15]. The finding is similar to the study done in Saudi Arabia [33], Europe [41], Spain [7], and South Korea [36]. Anemia in cancer patients with coexisting conditions is often caused by chronic inflammation, impaired erythropoiesis, nutritional deficiencies, and polypharmacy. Early screening and integrated care approaches, addressing cancer and comorbid diseases, along with nutritional and hematologic support, are recommended to improve treatment outcomes and patient quality of life.

The review found that hematologic cancer patients are 3.7 times more likely to develop anemia compared to those with other cancer types, indicating a strong association between cancer and anemia [19]. The finding is similar with study done in the United States [42], Austria [43], Italy [44], and South Korea [36]. The high prevalence of anemia in hematologic malignancies is attributed to factors such as bone marrow infiltration by malignant cells, chemotherapy‐induced myelosuppression, and chronic inflammation associated with the disease. These factors disrupt normal red blood cell production and iron metabolism. Regular monitoring of hemoglobin levels is crucial for hematologic cancer patients, and early interventions such as erythropoiesis‐stimulating agents, iron supplementation, or blood transfusions can help manage anemia, improving treatment outcomes and patient quality of life.

The reviewed article emphasizes that chemotherapy‐related factors, specifically receiving six or more cycles of chemotherapy, were three times more likely to develop anemia among cancer patients who received treatment [17, 19]. This is in line with the study done in Thailand [5], the United States [29], China [45], and Europe [41]. The risk of anemia is heightened by chemotherapy agents’ cumulative myelosuppressive effects, which can impair bone marrow function and decrease red blood cell production, with certain regimens like platinum‐based therapies exacerbating this effect. Healthcare providers should monitor hemoglobin levels in cancer patients undergoing multiple chemotherapy cycles, particularly those with platinum‐based or toxic drugs, to prevent or manage chemotherapy‐induced anemia, improving patient outcomes and quality of life.

The reviewed article revealed that cancer patients with bone metastasis have a significantly higher risk of developing anemia, with patients with bone metastases having nearly three times higher odds of developing anemia compared to those without metastasis [19], which is similar to the study findings in the United States [29], Thailand [5], Europe [41], and China [45]. Patients with metastatic cancer face an increased risk of anemia due to bone marrow infiltration, inflammation, increased hepcidin levels, and treatments like chemotherapy and radiotherapy, which hinder red blood cell production and iron utilization. Early anemia screening, regular monitoring, erythropoiesis‐stimulating agents, and iron supplementation are crucial for bone metastasis patients, with personalized treatment plans improving outcomes and quality of life.

The reviewed article indicates that cancer patients with a history of bleeding are 3.6 times more likely to develop anemia compared to those without such a history [13]. This finding aligns with studies from Saudi Arabia [6], Italy [46], and China [47]. Tumor‐related bleeding increases the risk of anemia due to blood loss, iron depletion, disrupted erythropoiesis, and compensatory mechanisms like elevated hepcidin levels. Regular monitoring of hemoglobin levels in cancer patients with a bleeding history is crucial for managing anemia, with interventions including iron supplementation, erythropoiesis‐stimulating agents, and appropriate treatments like surgery or hemostatic therapies.

### 4.1. Strengths and Limitations

This systematic review and meta‐analysis is the first comprehensive synthesis to estimate the national pooled prevalence of anemia and its associated factors among cancer patients in Ethiopia, incorporating data from multiple regions and healthcare settings. The study followed rigorous PRISMA guidelines, applied a registered protocol, and employed robust statistical methods, including sensitivity analysis and assessment of publication bias, to ensure the reliability and validity of findings. Some associated factors, including gender, age, and nutritional status, were derived from a limited number of studies, which may restrict the generalizability of these findings and warrant cautious interpretation. However, most included studies were cross‐sectional, limiting causal inference. Additionally, a major limitation of this meta‐analysis is that the heterogeneity in anemia definitions, diagnostic criteria, and cancer types across studies may have introduced heterogeneity in anemia definitions across the included studies. Because of the limited number of studies in each subgroup, we were unable to perform subgroup meta‐analyses to explore this variability further, and the pooled prevalence estimate should be interpreted cautiously. Finally, the reliance on secondary data from institutional records may have led to underreporting or incomplete data on key variables.

## 5. Conclusion and Recommendation

This systematic review and meta‐analysis revealed that anemia is a highly prevalent and under‐recognized complication among cancer patients in Ethiopia, affecting nearly two out of five individuals undergoing treatment. Several modifiable factors, such as advanced disease stage, poor nutritional status, chemotherapy exposure, and comorbid conditions, were significantly associated with anemia. These findings highlight the urgent need for integrating routine anemia screening, early diagnosis, and targeted interventions into oncology care pathways. To improve patient outcomes and quality of life, national cancer treatment guidelines should incorporate standardized anemia management protocols, enhance provider training, and strengthen access to essential diagnostic and therapeutic resources. Further longitudinal and interventional studies are also recommended to explore causal relationships and evaluate the effectiveness of anemia interventions in the Ethiopian context.

## Author Contributions

Sadik Abdulwehab and Frezer Kedir contributed substantially to the conception and design of the study, development of the protocol, literature search, data extraction, data analysis, and drafting of the manuscript. Aliyi Benti Daba, Dawit Tesfaye Rundasa, Ashenafi Tesfaye Yadesa, and Teferi Babu Itana contributed to data screening, quality appraisal of included studies, data validation, and critical revision of the manuscript for important intellectual content. Mesfin Dabasa Jigi, Yohannes Godie Ashebir, and Mathewos Mekonnen Gemmech contributed to methodological refinement, interpretation of findings, statistical verification, and manuscript editing. Sadik Abdulwehab and Frezer Kedir also prepared Tables 1, 2, and 3 and Figures 1–5. All authors critically reviewed the manuscript and agreed to be accountable for all aspects of the work. Sadik Abdulwehab had full access to all of the data in this study and takes complete responsibility for the integrity of the data and the accuracy of the data analysis. All authors critically reviewed, edited, and approved all final contents and take full responsibility for the accuracy and integrity of the manuscript.

## Funding

The authors have nothing to report.

## Disclosure

All authors have read and approved the final version of the manuscript.

## Ethics Statement

The authors have nothing to report.

## Consent

The authors have nothing to report.

## Conflicts of Interest

The authors declare no conflicts of interest.

## Supporting Information

Additional supporting information can be found online in the Supporting Information section.

## Supporting information


**Supporting Information** Supporting Appendix 1 contains the detailed search strategies, databases searched, search limits, and eligibility criteria used in this systematic review.

## Data Availability

The data that support the findings of this study are available from the corresponding author upon reasonable request.

## References

[1] Sung H. , Ferlay J. , Siegel R. L. et al., Global Cancer Statistics 2020: GLOBOCAN Estimates of Incidence and Mortality Worldwide for 36 Cancers in 185 Countries, CA: A Cancer Journal for Clinicians. (2021) 71, no. 3, 209–249, 10.3322/caac.21660. 33538338

[2] Memirie S. T. , Habtemariam M. K. , Asefa M. et al., Estimates of Cancer Incidence in Ethiopia in 2015 Using Population-Based Registry Data, JGO. (2018) no. 4, 1–11, 10.1200/JGO.17.00175. PMC6223441 30241262

[3] Aapro M. , Beguin Y. , Bokemeyer C. et al., Management of Anaemia and Iron Deficiency in Patients with Cancer: ESMO Clinical Practice Guidelines, Annals of Oncology. (2018) 29, iv96–110, 10.1093/annonc/mdx758. 29471514

[4] Gilreath J. A. and Rodgers G. M. , How I Treat cancer-associated Anemia, Blood. (2020) 136, no. 7, 801–813, 10.1182/blood.2019004017. 32556170

[5] Muthanna F. M. S. , Karuppannan M. , Abdulrahman E. , Uitrakul S. , Rasool B. A. H. , and Mohammed A. H. , Prevalence and Associated Factors of Anemia Among Breast Cancer Patients Undergoing Chemotherapy: a Prospective Study, Advances in Pharmacological and Pharmaceutical Sciences. (2022) 2022, 7611733–7611739, 10.1155/2022/7611733. 35464620 PMC9023199

[6] Badheeb A. M. , Ahmed F. , Badheeb M. A. et al., Anemia Profiles in Cancer Patients: Prevalence, Contributing Factors, and Insights from a Retrospective Study at a Single Cancer Center in Saudi Arabia, Cureus. (2023) 15, no. 7, 10.7759/cureus.42400. PMC10446849 37621805

[7] Steegmann J. L. , Sánchez Torres J. M. , Colomer R. et al., Prevalence and Management of Anaemia in Patients with Non-myeloid Cancer Undergoing Systemic Therapy: a Spanish Survey, Clinical and Translational Oncology. (2013) 15, no. 6, 477–483, 10.1007/s12094-012-0953-5. 23263906 PMC3663988

[8] Merlini L. , Cartenì G. , Iacobelli S. et al., Anemia Prevalence and Treatment Practice in Patients with Non-myeloid Tumors Receiving Chemotherapy, Cancer Management and Research. (2013) 5, 205–214, 10.2147/CMAR.S45236. 23946669 PMC3739422

[9] Aynalem M. , Adem N. , Wendesson F. et al., Hematological Abnormalities Before and After Initiation of Cancer Treatment Among Breast Cancer Patients Attending the University of Gondar Comprehensive Specialized Hospital Cancer Treatment Center, PLoS One. (2022) 17, no. 8, 10.1371/journal.pone.0271895. PMC9359539 35939445

[10] Belachew S. A. , Erku D. A. , Mekuria A. B. , and Gebresillassie B. M. , Pattern of chemotherapy-related Adverse Effects Among Adult Cancer Patients Treated at Gondar University Referral Hospital, Ethiopia: a cross-sectional Study, DHPS. (2016) 8, 83–90, 10.2147/DHPS.S116924. PMC5153262 27994485

[11] Berta D. M. , Teketelew B. B. , Chane E. et al., Hematological Changes in Women with Cervical Cancer Before and After Cancer Treatment: Retrospective Cohort Study, Scientific Reports. (2024) 14, no. 1, 10.1038/s41598-024-75937-6. PMC11555053 39528538

[12] Kassie T. D. , Yimenu B. W. , Baye Temesgen G. , Shimelash R. A. , and Abneh A. A. , Differences in the Count of Blood Cells Pre-and Post-chemotherapy in Patients with Cancer: a Retrospective Study (2022), Frontiers of Medicine. (2025) 12, 10.3389/fmed.2025.1485676. PMC12041017 40309726

[13] Kifle E. , Hussein M. , Alemu J. , and Tigeneh W. , Prevalence of Anemia and Associated Factors Among Newly Diagnosed Patients with Solid Malignancy at Tikur Anbessa Specialized Hospital, Radiotherapy Center, Addis Ababa, Ethiopia, Advances in Hematology. (2019) 2019, 1–8, 10.1155/2019/8279789. PMC6855075 31781226

[14] Setegn A. , Nakie G. , Yilkal Abebaw W.1 , Ferede Zegeye1 A. , and Abebe Gebrehana2 D. , 2024.

[15] Wassie M. , Aemro A. , and Fentie B. , Prevalence and Associated Factors of Baseline Anemia Among Cervical Cancer Patients in Tikur Anbesa Specialized Hospital, Ethiopia, BMC Women’s Health. (2021) 21, no. 1, 10.1186/s12905-021-01185-9. PMC7831239 33494721

[16] Wassie Y. A. , Zegeye A. F. , Gebrehana D. A. et al., Baseline Anemia and Its Associated Factors Among Adult Cancer Patients at Northwest Amhara Regional State Referral Hospitals, Northwest Ethiopia, 2021, Frontiers Oncology. (2024) 14, 10.3389/fonc.2024.1390052. PMC11250537 39015487

[17] Woldemariam A. G. , Tsehaye A. , Mokonen W. et al., Prevalence and Associated Factors of Anemia Among People with Cancer in ACSH, Tigray, Ethiopia [Internet], 2023, https://www.researchsquare.com/article/rs-3208295/latest.

[18] Wondimneh B. , Anekere Dasappa Setty S. , Gebregzabher Asfeha G. , Belay E. , Gebremeskel G. , and Baye G. , Comparison of Hematological and Biochemical Profile Changes in Pre-and Post-chemotherapy Treatment of Cancer Patients Attended at Ayder Comprehensive Specialized Hospital, Mekelle, Northern Ethiopia 2019: A Retrospective Cohort Study, 2021, Cancer Management and Research, 625–632. 10.2147/CMAR.S274821 PMC7837543 33519241

[19] Wondm S. A. , Dagnew S. B. , Gubae K. , Tesfaye T. C. , and Tamene F. B. , Determinants of Anemia Among Patients Receiving Cancer Chemotherapy in Northwest Ethiopia, Frontiers of Medicine. (2024) 11, 10.3389/fmed.2024.1415877. PMC11269183 39055698

[20] Nancy A. , Most Cancer Patients Don’T Receive Recommended Anemia Care, 2024, https://www.medscape.com/viewarticle/most-cancer-patients-dont-receive-recommended-anemia-care-2024a1000bxh.

[21] Hana A. , Anemia and Cancer: Symptoms, Causes, and Complications, 2023, https://www.medicalnewstoday.com/articles/anemia-and-cancer?utm_source=chatgpt.com.

[22] Clarke H. and Pallister C. J. , The Impact of Anaemia on Outcome in Cancer, Clinical and Laboratory Haematology. (2005) 27, no. 1, 1–13, 10.1111/j.1365-2257.2004.00664.x. 15686502

[23] Willis T. A. , Neal R. D. , Walter F. M. , and Foy R. , Priorities for Implementation Research on Diagnosing Cancer in Primary Care: a Consensus Process, BMC Health Services Research. (2023) 23, no. 1, 10.1186/s12913-023-10330-z. PMC10683096 38012602

[24] Musuka H. W. , Iradukunda P. G. , Mano O. et al., Evolving Landscape of Sickle Cell Anemia Management in Africa: a Critical Review, Tropical Medicine and Infectious Disease. (2024) 9, no. 12, 10.3390/tropicalmed9120292. PMC11680351 39728819

[25] Negussie F. , Giru B. W. , Yusuf N. T. , and Gela D. , Psychological Distress and Associated Factors Among Cancer Patients in Public Hospitals, Addis Ababa, Ethiopia: a cross-sectional Study, BMC Psychology. (2023) 11, no. 1, 10.1186/s40359-023-01079-5. PMC9921361 36765415

[26] Page M. J. , McKenzie J. E. , Bossuyt P. M. et al., The PRISMA 2020 Statement: an Updated Guideline for Reporting Systematic Reviews, Systematic Reviews. (2021) 10, no. 1, 10.1186/s13643-021-01626-4. PMC8008539 33781348

[27] JBI Manual for Evidence Synthesis, 2024, JBI Manual for Evidence Synthesis-JBI Global Wiki, https://jbi-global-wiki.refined.site/space/MANUAL, [Internet].

[28] Yetman D. , Chemotherapy-Induced Anemia: Symptoms, 2022, https://www.healthline.com/health/cancer/chemotherapy-induced-anemia.

[29] Xu H. , Xu L. , Page J. H. et al., Incidence of Anemia in Patients Diagnosed with Solid Tumors Receiving Chemotherapy, 2010;2013, CLEP. (2016) 8, 61–71, 10.2147/CLEP.S89480. PMC4847604 27186078

[30] Li R. , Wang X. , Yu G. , Pan J. , and Wang J. , Prevalence of Cancer Anemia and Correlation Between Anemia Status and Survival Time in Chinese Patients with Gastric Carcinoma, Journal of Clinical Orthodontics. (2008) 26, no. 15_suppl, 10.1200/jco.2008.26.15_suppl.20647.

[31] Majumdar S. and Shet A. S. , Cancer-Related Anemia in Northeast India: Many Questions, Cancer Research, Statistics, and Treatment. (2021) https://journals.lww.com/crst/fulltext/2021/01000/cancer_related_anemia_in_northeast_india__many.46.aspx?utm_source=chatgpt.com.

[32] Edward Winstead , Cancer Treatment Side Effects Are More Common in Women-NCI [Cgvblogpost], 2022, https://www.cancer.gov/news-events/cancer-currents-blog/2022/cancer-treatment-women-severe-side-effects.

[33] Alghamdi A. H. , Niyaz R. I. , Al-Jifree H. , Khan M. A. , and Alsalmi L. , Prevalence of Anemia Among Gynecologic Cancer Patients Who Received Chemotherapy, Radiotherapy, or a Combination of Both at King Abdulaziz Medical City, Jeddah, Cureus. (2021) 13, no. 8, 10.7759/cureus.17613. PMC8483600 34646664

[34] Barrett-Lee P. , Bokemeyer C. , Gascón P. et al., Management of Cancer-Related Anemia in Patients with Breast or Gynecologic Cancer: New Insights Based on Results from the European Cancer Anemia Survey, The Oncologist. (2005) 10, no. 9, 743–757, 10.1634/theoncologist.10-9-743. 16249356

[35] Boennelykke A. , Jensen H. , Østgård L. S. G. et al., Cancer Risk in Persons with new-onset Anaemia: a Population-based Cohort Study in Denmark, BMC Cancer. (2022) 22, no. 1, 10.1186/s12885-022-09912-7. PMC9306185 35864463

[36] Oh T. K. and Song I. A. , Anemia May Increase the Overall Risk of Cancer: Findings from a Cohort Study with a 12-Year Follow-up Period in South Korea, Cancer Epidemiology, Biomarkers & Prevention. (2021) 30, no. 7, 1440–1448, 10.1158/1055-9965.EPI-20-1840. 33879452

[37] Cerullo F. , Gambassi G. , and Cesari M. , Cancer-Related Anemia and Frailty in Older Persons, J Frailty Aging. (2012) 1, no. 3, 128–136, 10.14283/jfa.2012.21. 27093201

[38] Wang H. , Hua X. , Yao N. et al., The Urban-Rural Disparities and Associated Factors of Health Care Utilization Among Cancer Patients in China, Frontiers in Public Health. (2022) 10, 10.3389/fpubh.2022.842837. PMC8931518 35309211

[39] Lopes S. O. , Ribeiro S. A. V. , Morais D. de C. et al., Factors Associated with Anemia Among Adults and the Elderly Family Farmers, International Journal of Environmental Research and Public Health. (2022) 19, no. 12, 10.3390/ijerph19127371. PMC9224523 35742619

[40] Adamu A. L. , Crampin A. , Kayuni N. et al., Prevalence and Risk Factors for Anemia Severity and Type in Malawian Men and Women: Urban and Rural Differences, Population Health Metrics. (2017) 15, no. 1, 10.1186/s12963-017-0128-2. PMC5371260 28356159

[41] Barrett-Lee P. J. , Ludwig H. , Birgegård G. et al., Independent Risk Factors for Anemia in Cancer Patients Receiving Chemotherapy: Results from the European Cancer Anaemia Survey, Oncology. (2006) 70, no. 1, 34–48, 10.1159/000091675. 16493206

[42] Knight K. , Wade S. , and Balducci L. , Prevalence and Outcomes of Anemia in Cancer: a Systematic Review of the Literature, Americas Journal of Medicine. (2004) 116, no. Suppl 7A, 11S–26S, 10.1016/j.amjmed.2003.12.008. 15050883

[43] Steurer M. , Wagner H. , and Gastl G. , Prevalence and Management of Anaemia in Haematologic Cancer Patients Receiving Cyclic Nonplatinum Chemotherapy: Results of a Prospective National Chart Survey, Wiener Klinische Wochenschrift. (2004) 116, no. 11–12, 367–372, 10.1007/BF03040915. 15291288

[44] Paitan V. , Alcarraz C. , Leonardo A. et al., Anemia as a Prognostic Factor in Cancer Patients, Revista Peruana de Medicina Experimental y Salud Pública. (2018) 35, no. 2, 250–258, 10.17843/rpmesp.2018.352.3171. 30183919

[45] Cheng K. , Zhao F. , Gao F. et al., Factors Potentially Associated with chemotherapy-induced Anemia in Patients with Solid Cancers, Asian Pacific Journal of Cancer Prevention. (2012) 13, no. 10, 5057–5061, 10.7314/apjcp.2012.13.10.5057. 23244110

[46] Busti F. , Marchi G. , Ugolini S. , Castagna A. , and Girelli D. , Anemia and Iron Deficiency in Cancer Patients: Role of Iron Replacement Therapy, Pharmaceuticals. (2018) 11, no. 4, 10.3390/ph11040094. PMC6315653 30274354

[47] Bozzini C. , Busti F. , Marchi G. et al., Anemia in Patients Receiving Anticancer Treatments: Focus on Novel Therapeutic Approaches, Frontiers Oncology. (2024) 14, 10.3389/fonc.2024.1380358. PMC11018927 38628673

