# Supplementary material for: Prevalence and Risk Factors of Anemia Among Ethiopian Cancer Patients Undergoing Treatment: A Systematic Review and Meta‐Analysis
Source: Anemia. 2026 Jun 23;2026:7191757. doi: 10.1155/anem/7191757 (PMC13291442; doi:10.1155/anem/7191757)
Supplement: Supplementary file 1 — Supporting Information Supporting Appendix 1 contains the detailed search strategies, databases searched, search limits, and eligibility criteria used in this systematic review. [file ANEM-2026-7191757-s001.docx]

**Supplementary Appendix 1- Database Search Strings**

**PubMed**

("Anemia"[MeSH Terms] OR "anemia"[All Fields] OR "anaemia"[All Fields])

AND

("Cancer"[MeSH Terms] OR "cancer"[All Fields] OR "malignancy"[All Fields] OR "oncology"[All Fields] OR "neoplasm"[All Fields])

AND

("Prevalence"[All Fields] OR "burden"[All Fields] OR "epidemiology"[All Fields])

AND

("Associated factors"[All Fields] OR "risk factors"[All Fields] OR "determinants"[All Fields])

AND

("Ethiopia"[MeSH Terms] OR "Ethiopia"[All Fields])

**Scopus**

TITLE-ABS-KEY

((anemia OR anaemia)

AND

(cancer OR malignancy OR oncology OR neoplasm)

AND

(prevalence OR burden OR epidemiology)

AND

("associated factors" OR "risk factors" OR determinants)

AND

Ethiopia)

**Web of Science**

TS=(anemia OR anaemia)

AND

TS=(cancer OR malignancy OR oncology OR neoplasm)

AND

TS=(prevalence OR burden OR epidemiology)

AND

TS=("associated factors" OR "risk factors" OR determinants)

AND

TS=(Ethiopia)

**CINAHL (via EBSCOhost)**

(anemia OR anaemia)

AND

(cancer OR malignancy OR oncology OR neoplasm)

AND

(prevalence OR burden OR epidemiology)

AND

("associated factors" OR "risk factors" OR determinants)

AND

(Ethiopia)

**African Journals Online (AJOL)**

(anemia OR anaemia)

AND

(cancer OR malignancy OR oncology OR neoplasm)

AND

(prevalence OR burden)

AND

("associated factors" OR "risk factors")

AND

(Ethiopia)

**Google Scholar**

("anemia" OR "anaemia")

AND

("cancer" OR "malignancy" OR "oncology")

AND

("prevalence" OR "burden")

AND

("associated factors" OR "risk factors")

AND

"Ethiopia"

**Ethiopian University Institutional Repositories**

anemia AND cancer AND prevalence AND associated factors AND Ethiopia

**Manual Search**

Reference lists of all eligible articles and relevant review papers were manually searched to identify additional potentially eligible studies.

**Search Limits**

Language: English

Study setting: Ethiopia

Study type: Observational studies

Publication period: From database inception to May 03, 2025
